# Diverse and unconventional methanogens, methanotrophs, and methylotrophs in metagenome-assembled genomes from subsurface sediments of the Slate River floodplain, Crested Butte, CO, USA

**DOI:** 10.1128/msystems.00314-24

**Published:** 2024-06-28

**Authors:** Anna N. Rasmussen, Bradley B. Tolar, John R. Bargar, Kristin Boye, Christopher A. Francis

**Affiliations:** 1 Department of Earth System Science, Stanford University, Stanford, California, USA; 2 SLAC National Accelerator Laboratory, Menlo Park, California, USA; 3 Environmental Molecular Sciences Laboratory, Pacific Northwest National Laboratory, Richland, Washington, USA; 4 Oceans Department, Stanford University, Stanford, California, USA; China Agricultural University, Beijing, China

**Keywords:** subsurface, carbon, methane, metagenome-assembled genomes, floodplain

## Abstract

We use metagenome-assembled genomes (MAGs) to understand single-carbon (C1) compound-cycling—particularly methane-cycling—microorganisms in montane riparian floodplain sediments. We generated 1,233 MAGs (>50% completeness and <10% contamination) from 50- to 150-cm depth below the sediment surface capturing the transition between oxic, unsaturated sediments and anoxic, saturated sediments in the Slate River (SR) floodplain (Crested Butte, CO, USA). We recovered genomes of putative methanogens, methanotrophs, and methylotrophs (*n* = 57). Methanogens, found only in deep, anoxic depths at SR, originate from three different clades (*Methanoregulaceae*, *Methanotrichaceae*, and *Methanomassiliicoccales*), each with a different methanogenesis pathway; putative methanotrophic MAGs originate from within the Archaea (*Candidatus* Methanoperedens) in anoxic depths and uncultured bacteria (*Ca*. Binatia) in oxic depths. Genomes for canonical aerobic methanotrophs were not recovered. *Ca.* Methanoperedens were exceptionally abundant (~1,400× coverage, >50% abundance in the MAG library) in one sample that also contained aceticlastic methanogens, indicating a potential C1/methane-cycling hotspot. *Ca*. Methylomirabilis MAGs from SR encode pathways for methylotrophy but do not harbor methane monooxygenase or nitrogen reduction genes. Comparative genomic analysis supports that one clade within the *Ca*. Methylomirabilis genus is not methanotrophic. The genetic potential for methylotrophy was widespread, with over 10% and 19% of SR MAGs encoding a methanol dehydrogenase or substrate-specific methyltransferase, respectively. MAGs from uncultured *Thermoplasmata* archaea in the *Ca*. Gimiplasmatales (UBA10834) contain pathways that may allow for anaerobic methylotrophic acetogenesis. Overall, MAGs from SR floodplain sediments reveal a potential for methane production and consumption in the system and a robust potential for methylotrophy.

**IMPORTANCE:**

The cycling of carbon by microorganisms in subsurface environments is of particular relevance in the face of global climate change. Riparian floodplain sediments contain high organic carbon that can be degraded into C1 compounds such as methane, methanol, and methylamines, the fate of which depends on the microbial metabolisms present as well as the hydrological conditions and availability of oxygen. In the present study, we generated over 1,000 MAGs from subsurface sediments from a montane river floodplain and recovered genomes for microorganisms that are capable of producing and consuming methane and other C1 compounds, highlighting a robust potential for C1 cycling in subsurface sediments both with and without oxygen. Archaea from the *Ca*. Methanoperedens genus were exceptionally abundant in one sample, indicating a potential C1/methane-cycling hotspot in the Slate River floodplain system.

## INTRODUCTION

Riparian floodplains are productive and dynamic systems that contain high organic carbon (OC) and can be hotspots for geochemical cycling due to hydrological fluctuations. The OC within floodplains can be degraded through several mechanisms as floodplains may contain both oxic and anoxic sediments, depending on the hydrological conditions, thus producing a suite of low-molecular-weight and single-carbon (C1) compounds. These C1 compounds can have many fates, including being reduced to methane through methanogenesis or being oxidized through methylotrophy, which is defined as the ability to utilize C1 compounds (e.g., methane, methanol, and methylamine) as the sole carbon and energy source. When methane is the C1 compound that is oxidized, it is referred to specifically as methanotrophy. C1 generation and consumption are evolutionarily linked through the Wood-Ljungdahl pathway (WLP) (1).

Our understanding of the metabolic pathways and microorganisms responsible for both methanogenesis and methanotrophy is continually expanding, in large part due to the increasing sequencing of environmental samples. Methanogens are capable of reducing C1 compounds to methane gas through one of several different pathways, including using hydrogen (H_2_) and carbon dioxide (CO_2_) (hydrogenotrophic), acetate (aceticlastic), or other C1/methylated compounds (methylotrophic or hydrogen-dependent methylotrophic) [reviewed in reference (2)]. Methanogens are predominantly found within *Euryarchaeota* (*Halobacterota* and *Thermoplasmatota*) (3, 4) but have also been recently described in the *Candidatus* Bathyarchaeia (formerly *Bathyarchaea*, now in the *Thermoproteota*) (5) and *Verstraetearchaea* (6).

Our understanding of methanotrophy has expanded in recent years with methanotrophs now generally being divided into two groups, aerobic and anaerobic, based on their electron acceptors. Aerobic methanotrophs are found in the bacterial phyla *Proteobacteria* (7) and *Verrucomicrobia* (8, 9). Anaerobic methanotrophs include bacteria (10, 11), bacterial/archaeal consortia (12, 13), and archaea in the genus *Candidatus* Methanoperedens, which are capable of using various electron acceptors (14–17). Additionally, putative methanotrophy has been uncovered within the uncultivated bacterial lineage, *Candidatus* Binatia (formerly Binatota) (18). Methanotrophy requires a method for activating methane, either aerobically through a particulate methane monooxygenase (pMMO) or soluble methane monooxygenase (sMMO) enzyme or anaerobically through reverse methanogenesis. Activated methane can then enter several specialized methylotrophy pathways for carbon oxidation and assimilation that include methanol dehydrogenases, formaldehyde oxidation pathways, and formate dehydrogenases (19). Anaerobic methylotrophy can be carried out using three-component, corrinoid-dependent methyltransferase systems consisting of a substrate-specific methyltransferase (*mtxB*), a corrinoid-binding protein (*mtxC*), and a carbon-carrier methyltransferase (*mtxA*), with methanogens also using a reductive activase (*ramA*) (2, 20). These methyltransferase systems act on methylated compounds such as methanol, methoxylated compounds, methylamines, methylsulfides, and methanethiols. There are several different enzymes and pathways capable of carrying out each step in both the carbon oxidation and carbon assimilation required for methylotrophy, with many methylotrophs encoding some level of redundancy in carbon assimilation through either the ribulose monophosphate (RuMP), serine cycle and tetrahydrofolate pathway, or Calvin cycle (21), adding to the complexity of C1 cycling.

Here we use metagenome-assembled genomes (MAGs) to assess the genetic potential of microorganisms for methane cycling and broader methylotrophy in the Slate River (SR) floodplain. SR is a mountainous, high-elevation river located near Crested Butte (Gunnison County, CO, USA) and impacted by legacy mine activities for silver, lead, and zinc. The SR floodplain experiences seasonal rise and fall in the water table related to snowmelt-induced flooding, precipitation, and evapotranspiration. These fluctuations in the water table determine the inputs of nutrients and other substances to the subsurface and oxygen penetration depth, thus strongly influencing microbial communities and biogeochemical cycling. For example, water table height impacts lead speciation (22), iron-colloid composition (23), and zinc mobilization in the SR floodplain (24). Of particular relevance, the SR floodplain contains an oxic-anoxic interface within the fine-grained layer defined by the water table depth. We sampled six depths from 50 to 150 cm below sediment surface, capturing this transition between oxic and anoxic sediments in mid-June of 2018 when the water table height started to decline after reaching a peak in early June. During the sampling season (May–October 2018), water table height ranged from a peak in May of ~30 cm down to 105 cm in August then back up to ~50 cm in October (22). Seasonal variations in hydrology impacted porewater redox conditions throughout the sediment column. However, oxic conditions generally dominated above 90-cm depth and anoxic conditions below 110-cm depth, with redox conditions transitioning between 90 and 110 cm and seasonal, transient saturation occurring above 90 cm during high water conditions (22).

## RESULTS AND DISCUSSION

In total from the two locations and six different depths within the SR floodplain, we recovered 1,233 MAGs of >50% completeness and <10% contamination, spanning 38 different phyla based on the Genome Taxonomy Database (GTDB) taxonomy. We refer to MAGs based on their GTDB taxonomy throughout the text and reference former taxonomic names when relevant. The MAG library includes 135 archaeal MAGs and 1,098 bacterial MAGs, with the most MAGs originating from *Acidobacteriota* (*n* = 239), *Chloroflexota* (*n* = 148), and *Proteobacteria* (*n* = 157) (Fig. S1). After dereplication at 98% average nucleotide identity (ANI), the data set consisted of 683 representative MAGs. The dereplicated MAG library recruited between 13.2% and 38.3% of metagenomic reads in a sample (Table S1).

**Fig 1:**
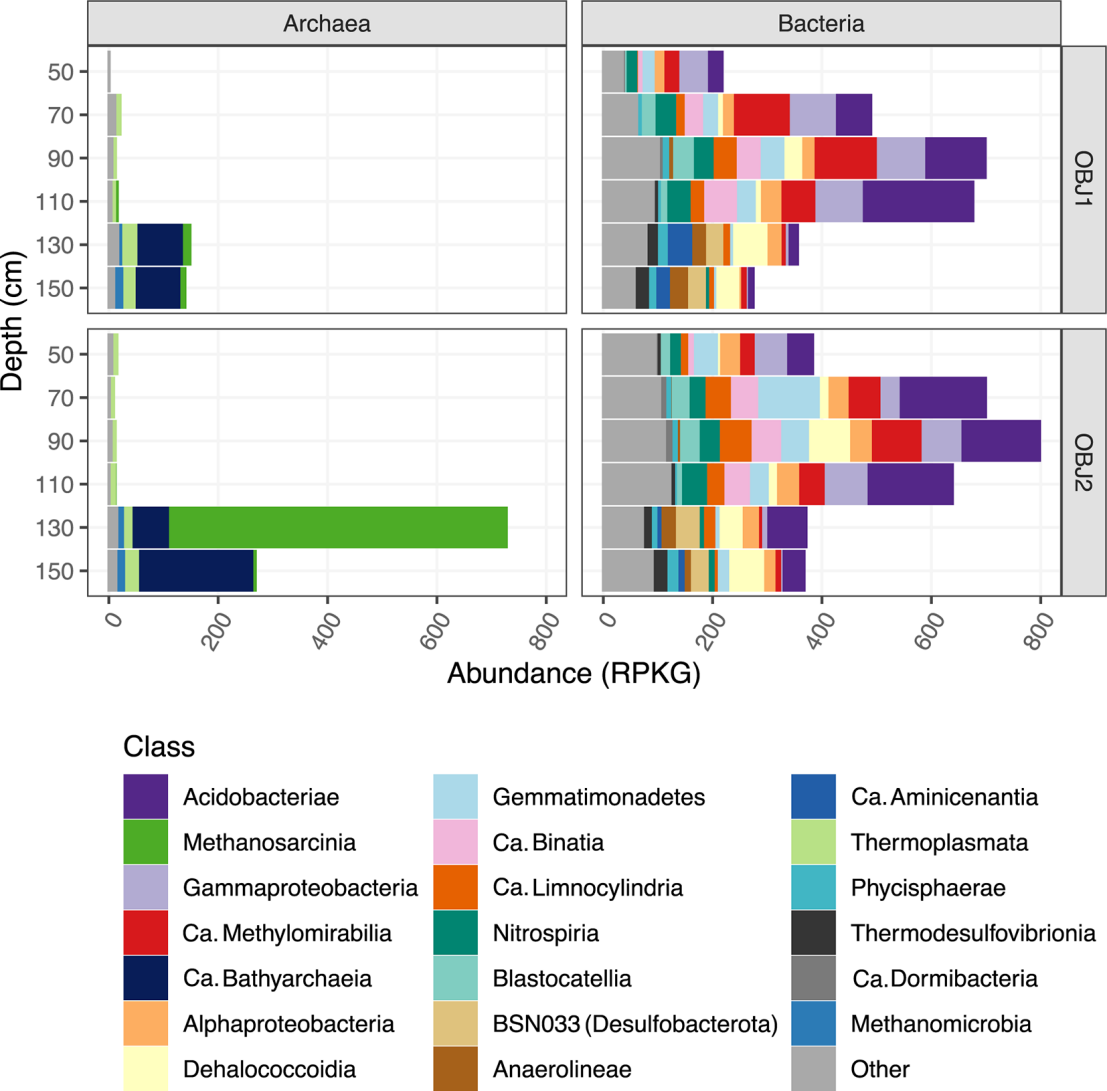
Abundance in reads per kilobase of genome per gigabase of metagenome (RPKG) of the 20 most abundant classes in the MAG library.

**Fig 2:**
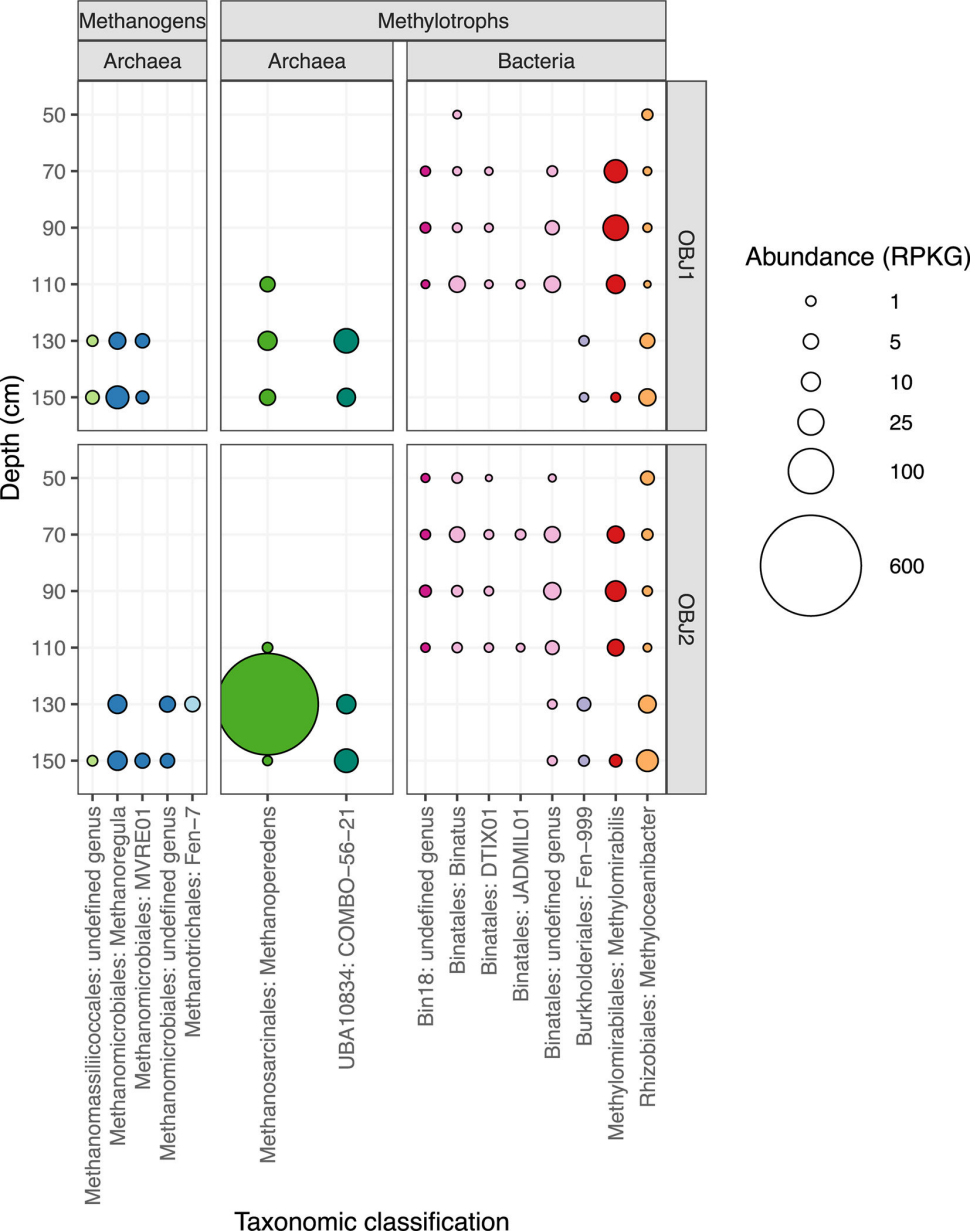
The abundance in reads per kilobase of genome per gigabase of metagenome (RPKG) of putative methane-cycling microorganisms and select methylotrophs. Points colored by MAG family and sized based on genus abundance. The *x*-axis denotes GTDB-assigned family and genus name, which does not include the *Candidatus* notation.

Even at the coarse level of phylum, we observed variations in the abundance of MAGs with depth, particularly between oxic and anoxic depths, similar to what we observed in 16S rRNA amplicon libraries from this site (B.B. Tolar, S. Kilpatrick, C. Dewey, S. Fendorf, J.R. Bargar, and C.A. Francis, submitted for publication). One obvious change in microbial communities is the dramatic increase in relative abundance of archaea in anoxic depths compared to oxic depths (Fig. 1). In contrast, abundant classes potentially containing methylotrophs such as *Gammaproteobacteria*, *Methylomirabilia*, and *Gemmatimonadetes* decrease between oxic and anoxic depths (Fig. 1). Archaea are most abundant and diverse in the anoxic depths (130 and 150 cm), with putative methane-cycling classes such as the *Methanosarcinia* (formerly within *Methanomicrobia*), *Methanomicrobia*, and *Ca*. Bathyarchaeia having high relative abundances in the community (Fig. 1). One MAG from the *Ca*. Methanoperedens genus is particularly abundant at OBJ2 in the 130-cm depth, indicating a possible methane-cycling hotspot. We identified MAGs as putative methane-cycling organisms (methanogens and methanotrophs) based on a combination of taxonomy and/or encoding methyl-coenzyme M reductase (MCR), pMMO, or sMMO genes. The distribution of putative methane-cycling MAGs was highly structured by depth in the soil column with a clear distinction between oxic and anoxic depths (Fig. 2). We obtained MAGs for 12 methanogens and 27 putative methanotrophs. We also recovered several other putative methylotroph MAGs, including those from the genera *Ca*. Methylomirabilis and *Methyloceanibacter*, highlighting the potential for cycling of other C1 compounds in addition to methane.

### Metabolic potential for methane production at depth

We recovered 12 methanogen MAGs from three orders, the *Methanomicrobiales* (*Halobacteriota*), *Methanotrichales* (*Halobacteriota*), and *Methanomassiliicoccales* (*Thermoplasmatota*). Within these three orders, MAGs originate from five different genera represented by nine lineages after dereplication at 98% ANI. Methanogen MAGs range from low to high quality (Table S2) and were only present in deep, saturated, anoxic depths (130 and 150 cm) that are conducive to methanogenesis (Fig. 2; Fig. S2).

We generated the most methanogen MAGs from within the *Methanomicrobiales* (*n* = 8) with varying quality levels (Table S2). *Methanomicrobiales* are known to carry out hydrogenotrophic (CO_2_-reducing) methanogenesis. Most *Methanomicrobiales* MAGs from SR contain some genes coding for the methyl and carbonyl branches of the WLP, MCR, MtrA-H, FdhAB, HdrABC, and MvhAGD, supporting that these organisms carry out hydrogenotrophic methanogenesis (Fig. S4). *Methanoregula* was the most abundant and diverse genus, and the *Methanoregulaceae* family was the predominant methanogen group in general (Fig. 2; Fig. S2). The relative abundance of different methanogen groups at SR indicates that hydrogenotrophic methanogenesis may dominate, but rates and relative contributions of different forms of methanogenesis require further study.

*Methanomassiliicoccales* are known to carry out H_2_-dependent methylotrophic methanogenesis growing on methanol, methylamines, or other methylated compounds using H_2_ as an electron donor for methanogenesis (3). The two MAGs generated from the family JACIVX01 (*Methanomassiliicoccales*) lack genes for the tetrahydromethanopterin (H_4_MPT) methyl branch of the WLP, most of the WLP carbonyl branch, and most genes for the Mtr enzyme as found in other *Methanomassiliicoccales* genomes (2, 25) while harboring genes for trimethylamine methyltransferase, HdrABC, and MvhAGD, indicating that these organisms carry out H_2_-dependent methylotrophic methanogenesis. These MAGs also contain cobalamin transport genes, which may be necessary for cobalamin-dependent proteins in the methylamine methyltransferase pathway, and the methyltransferase genes MTHFR and *folD* from the tetrahydrofolate (H_4_T) methyl branch of the WLP (Fig. S3). *Methanomassiliicoccales* are of low abundance in SR floodplains overall but found at both OBJ1 and OBJ2 at most anoxic depths (Fig. 2; Fig. S2). These organisms could be using methylamines with H_2_ generated from OC fermentation/degradation as opposed to fixing CO_2_ (2).

*Methanotrichaceae* (formerly *Methanosaetaceae*) perform aceticlastic methanogenesis (26–28). Both MAGs from the Fen-7 (*Methanotrichaceae*) genus are very high quality (Table S2) and contain a multitude of genes required for methanogenesis, including complete or near-complete pathways for the methyl and carbonyl branches of the WLP, genes for MCR, MtrA-H, Fpo, HdrDE, FdhAB, and HdrABC while lacking genes for Ech-H-ase. The genes present in SR MAGs provide support that these organisms carry out aceticlastic methanogenesis. *Methanotrichaceae* MAGs are of low abundance in SR and only found at OBJ2 in the 130-cm depth. This could indicate that conditions at this particular depth and location may be able to support aceticlastic methanogens. Intriguingly, this is also the depth where we see a high abundance of *Ca.* Methanoperedens (discussed in detail below).

Notably, none of the 57 *Ca*. Bathyarchaeia MAGs generated from SR floodplain contained genes annotated as MCR. *Ca*. Bathyarchaeia are diverse and abundant in SR floodplain sediments, and our recovered MAGs spanned a wide range of completeness (50.2%–98.13%, mean = 71.9%), which may have hampered our ability to identify genomes for methanogenic *Ca*. Bathyarchaeia. We also recovered several MAGs from clades within the *Thermoplasmata* sister to *Methanomassiliicoccales* that encoded some genes related to methanogenesis. However, they did not encode MCR or MtrA-H, aligning with findings in two recent studies (29, 30) and supporting that these organisms are unlikely to be methanogens. However, some of these MAGs encoded genes related to anaerobic methylotrophy and will be discussed in detail below.

### Anaerobic oxidation of methane by *Ca*. Methanoperedens

**Fig 3:**
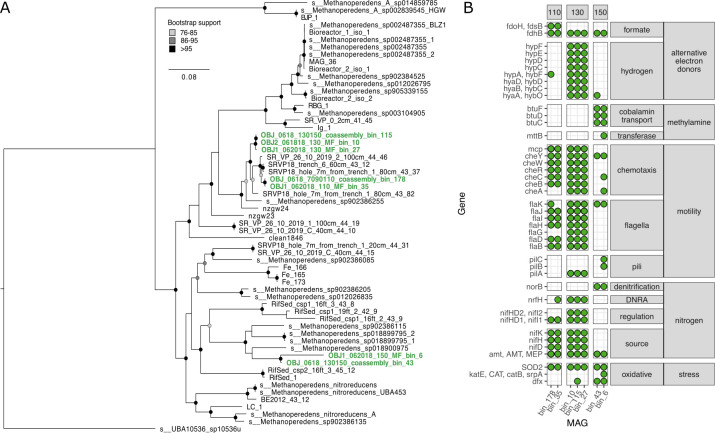
(A) Phylogeny of *Ca*. Methanoperedenaceae based on concatenated ribosomal protein tree of 32 ribosomal proteins using IQ-TREE with model LG + F + R4 with 100 bootstraps and midpoint rooted. Bootstrap support >75% indicated by node color. (B)Select genes of interest that vary between SR *Ca.* Methanoperedens MAGs, including formate dehydrogenase (*fdhB*, *fdoH/fdsB*), hydrogenase maturation proteins (*hypACDEF*), hydrogenase (*hya*/*hyb*), vitamin B12 transport system (*btuCD-F*), methyl-accepting chemotaxis protein (*mcp*), chemotaxis signal transduction proteins (*cheA*, *cheB*, *cheC*, *cheR*, *cheW*, and *cheY*), type IV pilus assembly protein (*pilABC*), flagellar proteins (*fla*), nitric oxide reductase (*norB*), cytochrome *c* nitrite reductase (*nrfH*), nitrogenase genes (*nifHDK*), ammonium transporter (*amt*), Fe-Mn family superoxide dismutase (SOD2), catalase (*katE*), and superoxide reductase (*dfx*).

We recovered seven (three after dereplication) high-quality (>90% completeness) MAGs belonging to the genus *Ca*. Methanoperedens, which contains microorganisms capable of anaerobic methane oxidation through reverse methanogenesis (13, 31). *Ca*. Methanoperedens can use a range of terminal electron acceptors during methanotrophy, including nitrate and metal oxides (such as iron and manganese), and have the genetic potential to use selenate, arsenate, and elemental sulfur (17, 32). *Ca*. Methanoperedens may also use formate as an electron donor (32, 33), further expanding their methylotrophic abilities. Some *Ca*. Methanoperedens also contain extra chromosomal elements (ECEs), including Borgs and other large ECEs (34, 35), that could contribute to even greater metabolic flexibility for this group. *Ca.* Methanoperedens in the SR floodplain are found in 110- to 150-cm depths (Fig. 1; Fig. S3). Our three representative MAGs have different depth distributions (Fig. S3) leading to “110-cm,” “130-cm,” and “150-cm” lineages with some variations between their genomic content (Fig. 3; Fig. S3). The shallower MAGs predominating at 110 and 130 cm fall into the same clade, while the “deep” MAGs (predominant at 150 cm) fall into another clade based on a concatenated ribosomal protein phylogeny (Fig. 3). *Ca.* Methanoperedens MAG OBJ_0618_130150_coassembly_bin_115 is of very high quality (100% complete, 0% contamination; Table S2) and reaches extremely high abundance in OBJ2 at 130-cm depth (~1,400× coverage), accounting for >50% of the MAG community (Fig. 1). *Ca.* Methanoperedens have been found to have a pleomorphic lifestyle, displaying both free-living and biofilm lifestyles (36). This massive abundance of *Ca*. Methanoperedens at OBJ2 could indicate a biofilm was formed at this depth and highlights one aspect of lifestyle flexibility that could allow these organisms to thrive in a dynamic floodplain environment.

*Ca*. Methanoperedens can couple methane oxidation to many different respiratory processes. MAGs in the “shallow” depths contain genes annotated as *nrfH* but lacked *nrfA*, indicating they may be capable of dissimilatory nitrate reduction to ammonia (DNRA) and may rely on nitrification products produced within shallower, unsaturated, oxic depths. These MAGs also contain genes for nitrogenase (*nif*) to fix nitrogen (Fig. 3). Shallow *Ca*. Methanoperedens in the SR floodplain also harbor many motility and chemotaxis genes (Fig. 3). *Ca*. Methanoperedens nitroreducens Verserenetto upregulates oxidative stress genes and flagellar genes upon exposure to oxygen (37). In a dynamic floodplain environment where water saturation and oxygen penetration depth vary, depending on snowmelt, evaporation, and precipitation, the potential for motility would likely benefit these organisms, especially at depths closer to the transition zone. Although all the SR *Ca*. Methanoperedens MAGs harbored *fdhB*, the 110-cm MAGs also harbored *fdoH*/*fdsB*, indicating formate, instead of methane, could serve as an electron donor (Fig. 3). Along these lines, MAGs from 130 cm encoded Ni-iron (Fe) hydrogenase and hydrogenase maturation genes that could potentially allow these organisms to use hydrogen as an electron acceptor (Fig. 3). Deep *Ca*. Methanoperedens have more oxidative stress genes, perhaps indicating a stronger susceptibility to oxidative stress and preference for more stable anoxic conditions. These MAGs also lacked DNRA and nitrogenase genes but did encode the trimethylamine-corrinoid protein co-methyltransferase gene (*mttB*) and cobalamin transporter BtuCD-F (Fig. S3). However, since the other corrinoid-dependent methyl transfer genes for methylamines were missing, it is unclear whether methylamines can be used as a substrate. Genes for other corrinoid-dependent methyltransferase systems using different methylated substrates were not present. A pangenomic comparison of the 11 genomes in the clade containing the shallow SR *Ca*. Methanoperedens and the 6 genomes in the deep clade identified several genes enriched in each group, including archaeal flagellin and nitrogenase in the shallow clade and catalase in the deep clade (Fig. 3).

Environmental data show that high levels of OC exist at OBJ2 at 150-cm depth (Fig. S4). It is possible that *Ca*. Methanoperedens are responding to increased methane produced from degradation of this OC. Intriguingly, ammonia is also highest at OBJ2 130 cm and nitrate is low (Fig. S3), although we cannot determine whether this could be a signal of DNRA by *Ca*. Methanoperedens versus other organisms or simply ammonia released during mineralization of OC. *Ca*. Methanoperedens are known to have many multiheme cytochromes (MHCs) that may allow them to use metals as electron acceptors (32, 38). MAGs from SR contain several cytochrome *c* biogenesis genes and MHCs and could be using metals such as iron or manganese as electron acceptors in the SR floodplain. Dissolved iron and manganese have a consistent peak at 130-cm depth at OBJ2 from June to October (22), indicating that metal-reducing conditions are generally favorable at this depth. Water table height peaked on 4 June 2018 and started to decline, but another transient rise in water table height occurred on 18 June 2018, when we sampled OBJ2 (22). Perhaps *Ca*. Methanoperedens responded to a transient rise in the water table and the resulting increased community fermentation of high dissolved organic carbon (DOC) at 150 cm. As previously mentioned, the aceticlastic methanogens are also only in the sample with high *Ca*. Methanoperedens abundance, indicating a potential increase in acetate availability also related to the increased OC at depth. Microbial communities can respond very rapidly to wetting events in this system and produce large pulses of carbon dioxide (B.B. Tolar, unpublished data).

### Aerobic methane oxidation by non-canonical methanotrophs

**Fig 4:**
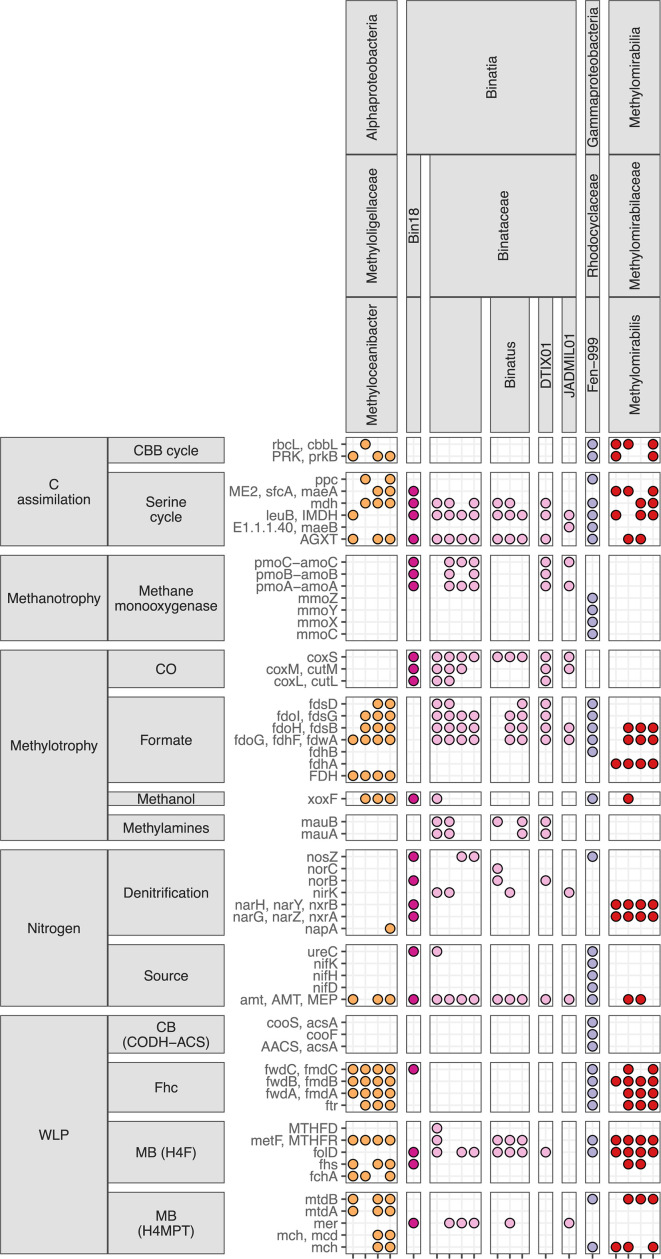
Select gene presence/absence for bacterial methylotrophs (including putative methanotrophs) sorted by GTDB-assigned taxonomy which does not include the *Candidatus* notation. CBB, Calvin-Benson-Bassham cycle; *rbcL*/*cbbL*, ribulose-bisphosphate carboxylase large chain; PRK/*prkB*, phosphoribulokinase; ME2/*sfcA*/*maeA*/*mdh*, malate dehydrogenase; *leuB*/IMDH, 3-isopropylmalate dehydrogenase; *mmo*, soluble methane monooxygenase; *pmo*, particulate methane monooxygenase; *cox*, carbon monoxide dehydrogenase; *fds/fdo/fdh* formate dehydrogenase; *xoxF*, lanthanide-dependent methanol dehydrogenase; *mauAB*, methylamine dehydrogenase; *nosZ*, nitrous oxide reductase; *nor*, nitric oxide reductase; *nirK*, nitrite reductase; *nar/nxr*, nitrate reductase/nitrite oxidoreductase; *napA*, nitrate reductase; *ureC*, urease; *nifHDK*, nitrogenase; *amt*, ammonium transporter; CODH-ACS, carbon monoxide dehydrogenase/acetyl-CoA synthase; *acsA*, acetyl-CoA decarbonylase/synthase, *cooFS*; anaerobic carbon-monoxide dehydrogenase; H4F, tetrahyrofolate; MTHFD, methylenetetrahydrofolate dehydrogenase; *metF*/MTHFR, methylenetetrahydrofolate reductase; *folD*, methylenetetrahydrofolate dehydrogenase; *fhs*, formate—tetrahydrofolate ligase; *fchA*, formate—tetrahydrofolate ligase; *mtd*, methylenetetrahydromethanopterin dehydrogenase; *mer*, 5,10methylenetetrahydromethanopterin reductase; *mch*, methenyltetrahydromethanopterin cyclohydrolase; Fhc, formyltransferase/hydrolase complex; *fwdABC/fmdABC,* formylmethanofuran dehydrogenase; *ftr*, formylmethanofuran-tetrahydromethanopterin N-formyltransferase.

Two (one after dereplication) high-quality Fen-999 (*Rhodocyclaceae*) MAGs encoding sMMO (Fig. 4) were recovered from anoxic depths. From oxic depths, we recovered several MAGs from the *Ca*. Binatia that contain genes annotated as *pmo* (Fig. 4). Intriguingly, we did not recover MAGs for canonical aerobic methanotrophs in *Alphproteobacteria*, *Gammaproteobacteria*, or *Verrucomicrobiae*. A handful of MAGs from these classes contained a single subunit for methane monooxygenase and intermittent downstream methylotrophy genes; however, their status as methanotrophs is unclear. We also recovered several MAGs from the *Ca.* Methylomirabilis and *Methyloceanibacter*, genera that contain methane-oxidizing organisms, though the SR MAGs did not encode methane monooxygenases (Fig. 4).

The Fen-999 MAG containing sMMO was present exclusively in anoxic depths. The MAG was of high quality (Table S2) and contained methylotrophy genes (Fig. 4), supporting the genetic potential for methanotrophy by this organism. While methanotrophs containing methane monooxygenases are considered aerobes due to the dependence of this enzyme on oxygen, a growing body of literature supports that methanotrophs and methane oxidation can occur under suboxic/anoxic conditions, possibly through cryptic O_2_ cycling [reviewed in reference (39)]. The genomes of these organisms support their methylotrophic potential, but their activity in anoxic depths must be confirmed. The soluble methane monooxygenase can also act on a variety of substrates in addition to methane (40), highlighting that this organism may be able to use other C1 compounds available in anoxic depths.

**Fig 5:**
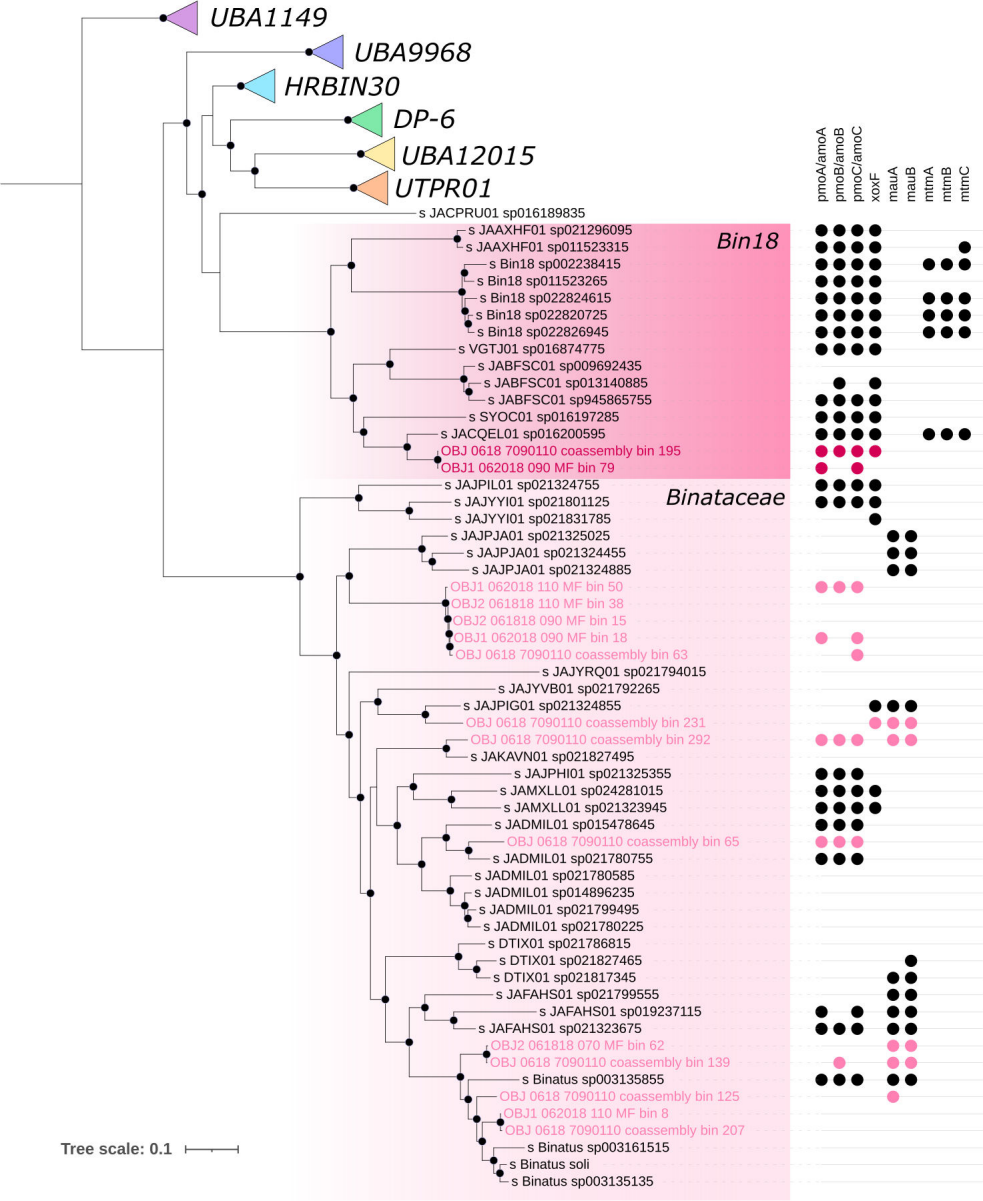
Phylogeny of GTDB species representative genomes from the *Ca*. Binatia based on 23 concatenated ribosomal genes made using IQ-TREE and model LG + F + R8 with 1,000 bootstraps. Branch nodes with >75% support shown with black dots. “Bin18*”* and *Ca*. Binataceae MAGs from SR highlighted in hot pink and light pink, respectively. Select relevant gene presence shown with points on the right, including particulate methane monooxygenase (*pmo*), lanthanide-dependent methanol dehydrogenase (*xoxF*), and methylamine dehydrogenase (*mauAB*) and monomethylamine corrinoid and methyltransferase genes (*mtmABC*). Labels denote the GTDB taxonomy which does not include the *Candidatus* notation.

Within the class *Ca*. Binatia (formerly the phylum *Ca*. Binatota, now within the *Desulfobacterota*_B in GTDB), several MAGs from the families “Bin18” and *Ca*. Binataceae contained putative pathways for methane oxidation (Fig. 4). These MAGs were generally most abundant in unsaturated, oxic depths (Fig. 2; Fig. S2). Many organisms from throughout the uncultured *Ca*. Binatia contain genes for methylotrophy based on one of the few previous studies investigating this group (18). We recovered 3 (1 after dereplication) Bin18 (*Ca*. Binatia) MAGs and 18 (9 after dereplication) *Ca*. Binataceae MAGs originating from at least 3 genera. MAGs from both of these families are putative methanotrophs based on gene annotations of *pmo* genes. Within the Bin18 (*Ca*. Binatia) family, nearly all genomes for species representatives in GTDB contain methanotrophy and methylotrophy genes such as methanol dehydrogenase (Fig. 5). However, within the *Ca*. Binataceae family, the genetic potential for methanotrophy and methylotrophy is patchy (Fig. 5). A phylogeny shows that the genes annotated as *pmoA* from the *Ca*. Binatia fall into clades distinct from known methanotrophs and closer to proposed butane monooxygenases (Fig. S5). It is unclear exactly what substrate these enzymes may be oxidizing, though the genetic potential for methylotrophy in many of these organisms support that methane or other alkanes could be substrates for their metabolism. *Ca*. Binatia MAGs from SR also harbored genes for alkane degradation (Fig. S6), as previously reported (18). We did not observe genes encoding other corrinoid-dependent methyltransferase systems outside of those acting on methylamines.

Though there is very limited information on the activity and metabolism for the uncultured lineage *Ca*. Binatia, a metaproteomics study from Columbia River sediments supports that some members of this lineage can use carbon monoxide as a supplemental energy and/or carbon source (41). *Ca*. Binatia did not express carbon fixation pathways but did express genes for aerobic oxidation of carbon monoxide, organic nitrogen mineralization, and denitrification in metaproteomes, highlighting an active role in carbon and nitrogen cycling in the hyporheic zone (41). These Columbia River sediments from the hyporheic zone did not harbor any methanotrophs or methanogens, unlike in the SR floodplain sediments presented here. The diversity of this uncultured clade and of the putative methanotrophs in SR floodplain sediments is intriguing. The putative methylotrophic *Ca*. Binatia MAGs contained genes for denitrification and other metabolisms (Fig. S6), highlighting the need for further culturing, biochemical, and *in situ* activity studies to confirm the proposed metabolisms for the *Ca*. Binatia and their activity in the environment.

**Fig 6:**
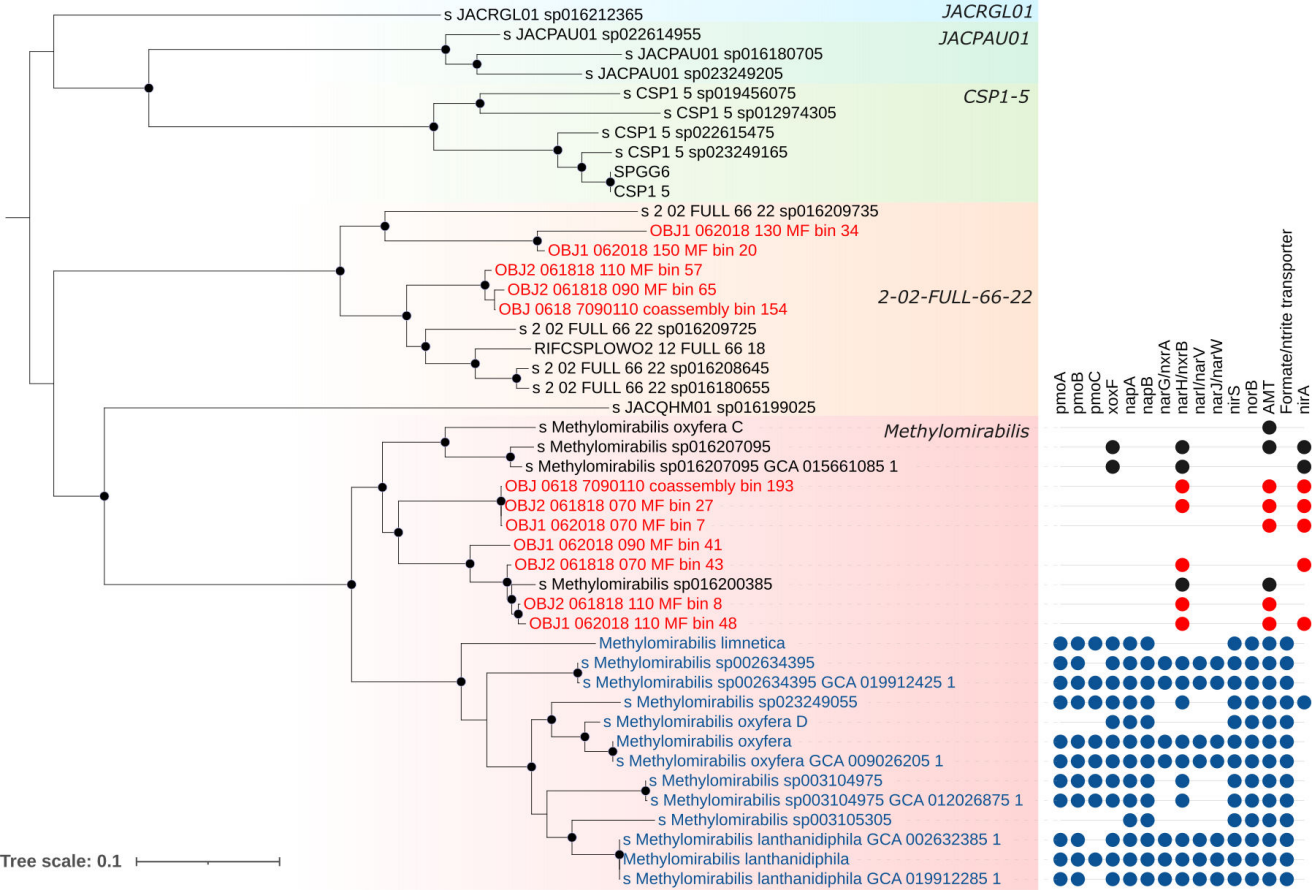
Phylogeny of *Ca*. Methylomirabilales based on concatenated ribosomal protein tree of 22 ribosomal proteins using IQ-TREE with model JTT + F + R4 with 1,000 bootstraps and midpoint rooted. Branch nodes with bootstrap support >75% shown by black dots. Blue indicates methanotrophic lineages within the *Ca*. Methylomirabilis, and red indicates MAGs from SR. Gene presence for the two *Ca*. Methylomirabilis genus clades shown to the right with methanotrophic lineage shown in blue, SR MAGs shown in red, and other methylotrophic genomes shown in black. Labels denote the GTDB taxonomy which does not include the *Candidatus* notation. *pmo*, particulate methane monooxygenase; *xoxF*, lanthanide-dependent methanol dehydrogenase; *nap*, nitrate reductase; *nar/nxr*, nitrate reductase/nitrite oxidoreductase; *nor*, nitric oxide reductase; *nirS* nitrite reductase (dissimilatory); amt, ammonium transporter; *nirA*, ferredoxin-nitrite reductase (assimilatory).

Our results yielded several MAGs from both the *Ca*. Methylomirabilis and *Methyloceanibacter* genera. Although both genera contain several strains capable of methanotrophy, SR MAGs from these groups do not appear to be methanotrophs (Fig. 4). *Ca*. Methylomirabilis is best known for containing organisms capable of nitrite-dependent anaerobic methane oxidation (n-DAMO). *Ca*. Methylomirabilis are abundant in oxic depths and yielded 12 (4 after dereplication) MAGs from 50.4% to 91.6% completeness (Table S2). However, SR MAGs lack PMO, *nirS*, Nod, *napA*, and *norB* but do harbor genes for NAR/NXR. Phylogenomic and pangenomic analyses reveal that the SR *Ca*. Methylomirabilis fall into a clade of the genus where no genomes thus far contain PMO or relevant denitrification genes (Fig. 6). It is unlikely the *Ca*. Methylomirabilis in SR are performing n-DAMO or methane oxidation; however, MAGs do contain methanol dehydrogenase and further downstream genes for methylotrophy, supporting their role in C1-cycling as methylotrophs (Fig. 4). Similarly, all eight *Methyloceanibacter* MAGs from SR lack methane monooxygenases and are not closely related to the known methanotrophic species but do contain methylotrophy genes (Fig. 4; Fig. S7). In the SR floodplain, *Methyloceanibacter* are found at all depths with representative MAG abundance partitioned by depth (Fig. 2; Fig. S2).

Four other MAGs encoded a single methane monooxygenase subunit, however, their genome completeness (range = 51%–93%) may have hindered capturing the entire methane oxidation pathway. Although most contained a smattering of downstream methylotrophy genes (Fig. S8), we excluded them from our analysis. Generally, these MAGs were of low abundance [~2 reads per kilobase of genome per gigabase of metagenome (RPKG)] but could highlight the functional redundancy of methane oxidizers in this dynamic system. It is possible that, as environmental conditions change, these organisms could become more prominent methane oxidizers. Amazon River floodplain sediments contain diverse and abundant methanotrophs (42) and methanogens, leading to robust methane cycling with floodplains acting as both a methane sink or source, depending on flooding (43). Interestingly, in Amazon River floodplains, methanogens and methanotrophs were resilient to dramatic environmental fluctuations (i.e., flooding) and community structure was more strongly shaped by soil physicochemical factors than flooding (42, 43). Further study of methane fluxes as well as the activity of microorganisms in the SR sediments will shed light on the production and consumption of methane in the system.

### Widespread genetic potential for methylotrophy

Although the genetic potential for methanotrophy was limited to a select few groups, consumption of other C1 compounds may be important in SR floodplain sediments. A survey of all MAGs containing an annotated methanol dehydrogenase gene revealed widespread potential for methylotrophy in the classes *Alphaproteobacteria*, *Gammaproteobacteria*, *Gemmatimonadetes*, and *Ca*. Methylomirabilia that were predominantly found in oxic soils (Fig. S9). In total, 10% of the 1,233 MAGs at SR contained methanol dehydrogenase. MAGs containing methanol dehydrogenase typically also contained the methyl branch of WLP and other downstream pathways necessary for methylotrophy (Fig. S10). Similarly, a study from nearby East River floodplain sediments found methanol dehydrogenase in ~10% of MAGs (44). Methanol is produced by pectin and lignin degradation and is a major volatile organic compound produced by plants and released into the atmosphere (40, 45). Methanol-oxidizing methylotrophs in sediments are important for mediating methanol fluxes to the atmosphere. Indeed, methanol oxidation can be robust in soils. A study of California grassland subroot (10- to 40-cm depth) soils found methanol dehydrogenase to be the most abundant protein in the proteome, predominantly from *Gemmatimonadetes* and also *Ca*. Rokubacteria (*Ca*. Methylomirabilia) (46), and methanol dehydrogenase genes (along with formate oxidation genes) were highly expressed in sediments of East River (44), a neighboring watershed to Slate River, CO, USA. Methanol-specific corrinoid-dependent methyltransferase genes were more rare, with methanol co-methyltransferase, *mtaB*, occurring in 1.6% of MAGs.

Methylamine dehydrogenase genes *mauA* or *mauB* were found in 14 genomes, primarily from *Ca*. Binatia and *Gammaproteobacteria*, while the dimethylamine/trimethylamine dehydrogenase gene *dmd-tmd* was found in 11 bacterial MAGs. Outside of methanol-specific genes, all other genes encoding corrinoid-dependent methyltransferase systems associated with anaerobic methylotrophy utilized methylated amines, including trimethylamine (*mttB* and *mttC*), dimethylamine (*mtbC*), monomethylamine (*mtmB*), and glycine-betaine (*mtaA* and *mtgB*). These genes were found in *Ca*. Bathyarchaiea and *Thermoplasmata* as discussed below, as well as a diversity of bacterial groups, most commonly within the *Acidobacterota* (particularly within *Acidobacteriae* and *Candidatus* Aminicenantia) and *Chloroflexota* (particularly within *Anaerolineae* and *Candidatus* Limnocylindria). The trimethylamine-corrinoid methyltransferase gene, *mttB*, was most commonly annotated, being present in 17% MAGs. In total, genes encoding for a substrate-corrinoid methyltransferase were present in 19.4% of MAGs. Genes encoding other corrinoid-dependent methyltransferase systems acting on methoxylated (*mtv* and *mto*), methylated sulfur (*mts* and *mtp*), and halogenated (*cmuA*, *cmuB*, and *dcmA*) compounds were not annotated in any MAGs. The widespread genetic potential for methylotrophy highlights the importance of OC breakdown and C1 cycling in this system under both oxic and anoxic conditions.

### Potential for methylotrophic acetogenesis in thermoplasmata clade *Ca*. Gimiplasmatales

Not only did we uncover widespread potential for aerobic methylotrophy in SR, but also we identified potential anaerobic methylotrophy in non-methanogenic archaeal groups. Anaerobic methylotrophs include methylotrophic methanogens, acetogenic bacteria, and sulfate-reducing bacteria, with the latter two groups generally using methyltransferase systems, H_4_F and the WLP for energy conservation (47–49). In SR, we recovered MAGs from several clades within the *Thermoplasmata* that are sister to the methanogenic order, *Methanomassiliicoccales*. Previous studies of MAGs from *Thermoplasmata* have found several basal clades to *Methanomassiliicoccales* that lacked MCR genes but contained a handful of genes related to methanogenesis, including HdrA-D, MvhAGD, and FmdE, with a few also encoding *mttB*, *mttC*, and *mtmB* (29, 30). We obtained MAGs from these sister orders, including *Ca*. Gimiplasmatales (UBA10834) and RBG-16–68-12, that did not encode MCR but encoded HdrA-D, MvhAGD, FmdE, most of the H_4_F methyl branch (Fhs, FolD, and MTHFR) of the WLP, and a handful also encoded *mttB* (Fig. S11). We obtained five MAGs from within the genus “COMBO-56–21” in *Ca*. Gimiplasmatales (29) containing the aforementioned genes and also including *acsA-E* for the carbonyl branch of WLP (as seen in methylotrophs), an acetate-CoA ligase (*acdAB*) (50), the RuMP pathway, rGlyP (51), complete pathways for glycolysis/glucogenesis, the pentose phosphate pathway, isoprenoid synthesis, *de novo* nucleotide synthesis, and amino acid synthesis. The genetic repertoire of these MAGs indicates these organisms could be capable of methylotrophic acetogenesis. Anaerobic methylotrophy has been proposed for archaea within the *Ca*. Brockarchaeota*,* which have been hypothesized to perform anaerobic C-cycling in hot springs and mesophilic sediments by using the H_4_F methyl branch and a rGlyP pathway (52). *Ca*. Methanoperedens can produce acetate during anaerobic methanotrophy (50, 53, 54). Others have also proposed methylotrophic acetogenesis in some clades of *Ca*. Bathyarchaeia based on the presence of methylamine transferases and WLPs (55). The possibility of methylotrophy in *Ca*. Gimiplasmatales has also been proposed previously (29), though not necessarily linked to acetogenesis.

Acetogenesis has been described in several groups of archaea, including *Ca*. Bathyarchaeia (56), Asgard archaea (57, 58), and methane-based acetogenesis in ANME (27, 50, 54, 59). The SR MAGs from the genus “COMBO-56–12” (*Ca*. Gimiplasmatales) encoded genes for the methylation of trimethylamines (*mttB* and *mttC*), methylamine (*mtmB*), and glycine betaine (*mtgB*) (Fig. S11), supporting that trimethylamine utilization may occur in *Ca*. Gimiplasmatales as previously suggested (29) as well as the use of other methylated amines. Given the lack of the second methyltransferase genes for these three-component, corrinoid-dependent methyltransferase systems, the transfer of the methylated compound from the corrinoid protein to tetrahydrofolate may occur via an undescribed protein and proceed through the H_4_F pathway to join the rGlyP or RuMP, as proposed for *Ca*. Brockarchaeota (52). Compounds can also make it through the RuMP to the non-oxidative pentoses phosphate pathway and ultimately to pyruvate and acetyl-CoA. ATP could be generated via the acetate-CoA ligase (60). The presence of HdrABC and MchAGD could recycle CoM. The most complete (97.6%) COMBO-56–21 MAG contains complete pathways for *de novo* purine and pyrimidine biosynthesis; threonine, valine, and isoleucine biosynthesis; thiamine biosynthesis; and isoprenoid biosynthesis; as well as the complete pathway for PRPP biosynthesis. This MAG also contains a near-complete gluconeogenesis pathway minus the step for glucose-6-phosphate isomerase, as seen in an analysis of several *Ca*. Gimiplasmatales (29). This could indicate that gluconeogenesis participates in the PPP shunt, as the final step to glucose is missing. There is also an incomplete tricarboxylic acid (TCA) cycle (only the second carbon oxidation half from isocitrate to malate). The COMBO-56–21 MAGs were only present in anoxic depths and are abundant in SR (Fig. 2; Fig. S2). These COMBO-56–21 also harbored motility genes (Fig. 3). A pangenome of available COMBO-56–21 genomes revealed an average genome size of 1.9 Mb (for genomes >90% complete). Other archaeal MAGs in *Thermoplasmata*, found predominantly at oxic depths, may be capable of carbon monoxide oxidation based on the presence of *coxMSL* (Fig. S11). Our understanding of the metabolic diversity of *Thermoplasmata* has greatly expanded in recent years to include diverse non-methanogenic metabolisms in several candidate orders, including fermentation in SG8-5 (61), copper membrane monooxygenases of unknown function in *Ca*. Angelarchaeales (62), acidophilic heterotrophy in the *Ca*. Lutacidiplasmatales (63), and mixotrophy in *Ca*. Gimiplasmatales (29). Our study builds on these findings to highlight that these abundant sediment organisms may also be methylotrophs.

### SR *Ca*. Bathyarchaeia MAGs lack genes for methanogenesis

*Ca*. Bathyarchaeia are known for anaerobic OC cycling and processing complex carbohydrates (64, 65), with some clades containing the metabolic potential for methanotrophy (5), methylotrophy (55), and acetogenesis (56). However, their potential roles as methylotrophs in SR floodplain sediments were less clear. Although none of the *Ca*. Bathyarchaeia MAGs from the SR floodplain harbored MCR, most contained near-complete or complete WLPs and some genes found in methanogenesis and methylotrophic pathways (Fig. S11). Some *Ca*. Bathyarchaeia MAGs from SR encoded partial corrinoid-dependent methylamine transfer systems, most commonly, *mttB*, or methanol dehydrogenases in addition to genes for versatile C metabolisms, suggesting they could be facultative methylotrophs. Even if the *Ca*. Bathyarchaeia themselves are not carrying out methylotrophy, many SR *Ca*. Bathyarchaeia MAGs encoded genes for fermentation and aromatic carbon degradation, indicating they could play an important role in OC degradation and supplying C1 compounds to methanogens and methylotrophs. *Ca*. Bathyarchaeia are most abundant at OBJ2 at 150-cm depth (Fig. 1), where OC is highest (Fig. S3), though they are generally abundant within all anoxic depths.

### Conclusions

Diverse methane-cycling microorganisms are found at the SR floodplain, including recently described groups such as *Ca*. Binatia, *Methanomassiliicoccales*, and *Ca*. Methanoperedens, the latter of which was exceptionally abundant in the anoxic 130-cm depth at OBJ2. Non-canonical methanotrophs were the dominant putative methanotrophs in the system, with canonical bacterial methanotrophs being notably absent from the MAG library. Surprisingly, *Ca*. Methylomirabilis in SR do not harbor pathways encoding for n-DAMO, and our analysis supports that other related strains in this genus are also not methanotrophs. A broader survey for methylotrophy revealed widespread potential for methanol oxidation in oxic depths and anaerobic methylotrophy, particularly utilizing methylated amines, in some abundant *Ca*. Bathyarchaeia, *Thermoplasmata*, and *Acidobacterota* in anoxic depths of the SR floodplain.

## MATERIALS AND METHODS

### Sample collection

Samples were collected at two sites, OBJ1 and OBJ2, from a gravel bed floodplain at the confluence of a tributary of the Slate River, Oh-Be-Joyful Creek, and Slate River (38°54′34.59′′ N, 107°1′43.40′′ W), at six different depth horizons including 50, 70, 90, 110, 130, and 150 cm below the sediment surface. Samples were collected on 18 June 2018 (OBJ2) and 20 June 2018 (OBJ1), during a historically low water year in the western USA.

### Environmental data

Environmental data were collected from our Slate River sites and measured as previously described in references (22, 24). Briefly, porewater was collected through 0.6-µm pore-size Rhizon samplers (Rhizosphere Research Products, Wageningen, Netherlands) at each soil depth horizon for dissolved metal and organic carbon quantification. Samples for dissolved metal quantification were acidified with nitric acid to ~2% final concentration of acid. Soil samples were collected with a bucket auger from depths corresponding to those of porewater rhizon samplers. Dissolved Fe and S were measured on a Thermo Scientific ICAP 6300 Duo View inductively coupled plasma optical emission spectrometer with a solid-state CID detector (Thermo Fisher Scientific). Solid-phase metals and sulfur were measured by X-ray fluorescence (XRF). Solids were dried and ground for XRF analysis. Additionally, aqueous phase concentrations of dissolved inorganic nitrogen (DIN), total OC (TOC), and total nitrogen (TN) were measured via water extraction by suspending 1 g of soil in 10 mL of ultrapure water, shaking at room temperature for 2 h, centrifuging at 4,000 rpm for 10–15 min, and filtering the aqueous phase through a 0.22-µm disk filter (Fisher Scientific). DIN (ammonia and nitrate) concentrations were measured on a SmartChem 200 Discrete Analyzer (WestCo Scientific Instruments, Brookfield, CT, USA) by spectroscopy. TOC and TN were measured by the Arizona Laboratory for Emerging Contaminants, at the University of Arizona, Tucson, AZ, USA (https://www.alec.arizona.edu) on a Shimadzu TOC-L_CSN_ (Shimadzu Scientific Instruments, Columbia, MD, USA).

### DNA extraction

DNA was extracted from frozen soil/sediment samples using the DNeasy PowerMax Soil Kit (Qiagen). Each soil sample was thawed, and a subsample (~5 g) was weighed out directly into a PowerMax bead tube for immediate extraction. The manufacturer’s protocol was followed as written, with the addition of an incubation step (80°C for 40 min) to improve yield, immediately after bead beating (10-min vortex). After extraction, DNA was eluted in 5-mL solution C6 and subsequently concentrated to ~1 mL using VivaSpin Turbo 15 columns (Sartorius). DNA concentration (ng/µL) was determined using the Qubit dsDNA Broad Range Assay (Invitrogen) following the manufacturer’s protocol.

### Metagenomic assembly, binning, and annotation

Twelve metagenomes were sequenced via the Joint Genome Institute (JGI) through a FICUS project (proposal ID 504298) on an Illumina NovaSeq S4. Metagenome sizes and read counts are available in Table S1. Quality Controlled Filtered Raw metagenome data (JGI project IDs 1359525–1359536) were downloaded from JGI Genome Portal for assembly, binning, and refining using the metaWRAP (v.1.3.2) pipeline (66). Assemblies and co-assemblies were made using megahit. Single-sample assemblies were binned using contigs of >2,000 nt using MetaBAT2 (v.2.12.1) (67), MaxBin 2.0 (v.2.2.6) (68), and CONCOCT (v.1.0.0) (69) with multiple fastq files grouped by surface (50-cm depth), middle depth (70–110 cm), and anoxic depths (130–150 cm). Co-assemblies were binned in a similar manner using contigs of >2,500 nt in length and multiple fastq files. Bins were consolidated and filtered using *metawrap bin_refinement* to be above >50% completeness and have <10% contamination as calculated via CheckM (v.1.1.3) (70). All MAGs were dereplicated using dRep (v.3.1.1) (71) at 98% ANI. Taxonomic classification for each representative MAG was made using the Genome Taxonomy Database Toolkit (72) with the database release 07-RS207. Reads were competitively recruited to dereplicated MAG library using Bowtie2 (v.2.4.2) (73) and the default parameters. Abundances are displayed as unpaired reads recruited per genome size of MAG in kilobases of MAG divided by gigabase of metagenome (RPKG), and coverage values were calculated using inStrain (74). Genes were called using Prodigal (v.2.6.3) (75), and translated gene annotations were performed via KEGG using GhostKOALA (76). For select MAGs of interest, translated sequences were also annotated using the RAST tool kit (RAST-tk v.2.0) (77, 78) for SEED (79) annotation. Genomes were also annotated using the METABOLIC (80) pipeline.

### Phylogenomics and pangenomics

Genomes for species representatives for select clades in the GTDB database (release 07-RS207) were downloaded from the National Center of Biotechnology Information (NCBI) database. Additional *Ca*. Methanoperedens genomes were downloaded from NCBI. Anvi’o (hope v.7) (81) was used to annotate, analyze, and compare downloaded methane-cycler genomes, in addition to the finalized MAGs generated in this study, following the standard phylogenomics and pangenomics workflows. Contigs shorter than 500 bp in length were excluded from the analysis. Conserved ribosomal and housekeeping genes were annotated with the seven default hmm databases used by *anvi-run-hmms* with the argument *–also-scan-trnas*, which uses tRNAscan-SE (v.2.0.7) to annotate tRNAs. All genomes were annotated using COG, KEGG KO, and pfams through the anvi’o pipeline with Diamond set to fast. Completeness and contamination were calculated using CheckM (v.1.1.3). Concatenated and aligned amino acid sequences were retrieved from MAGs using *anvi-get-sequences-for-hmm-hits* and MUSCLE (82) for bacterial or archaeal ribosomal genes. Genomes had to include at least 50% of ribosomal genes, and ribosomal genes had to be found in >90% of genomes to be included in alignments. Gaps in amino acid alignments with less than 50% coverage were removed using trimAl (v.1.4.rev15) (83). Then IQ-TREE (v.2.0.3) (84) was used for extended model selection for phylogeny of MAGs via -m MFP with the final phylogenomic tree based on the best model and with 1,000 bootstraps. Gene enrichment between groups was calculated using the *anvi-compute-functional-enrichment* function, and the adjusted *P* value was used to correct for multiple testing (85). Gene sequences of interest (i.e., *pmoA*) were collected from pangenomes using *anvi-get-sequences-for-gene-clusters*. Gene summaries for pangenomes were generated using *anvi-summarize*.

### Figure generation

Figures were generated using R (v.4.1.2). Phylogenetic tree figures were edited using iTOL (v.6.8.2) and FigTree (v.1.4.4). Figures were formatted using Inkscape (v.1.3).

## Data Availability

The MAGs described in this study have been uploaded to figshare (10.6084/m9.figshare.25320592).

## SUPPLEMENTAL MATERIAL

Supplemental figures msystems.00314-24-s0001.docx Fig. S1- S11.

Table S1 msystems.00314-24-s0002.xlsx Metagenome data.

Table S2 msystems.00314-24-s0003.xlsx Taxonomic and genome quality data for C1-cycling MAGs.

ASM does not own the copyrights to Supplemental Material that may be linked to, or accessed through, an article. The authors have granted ASM a non-exclusive, world-wide license to publish the Supplemental Material files. Please contact the corresponding author directly for reuse.
