# Supplementary material for: Diverse and unconventional methanogens, methanotrophs, and methylotrophs in metagenome-assembled genomes from subsurface sediments of the Slate River floodplain, Crested Butte, CO, USA
Source: mSystems. 2024 Jun 28;9(7):e00314-24. doi: 10.1128/msystems.00314-24 (PMC11264602; doi:10.1128/msystems.00314-24)
Supplement: Supplemental figures — Fig. S1- S11. [file msystems.00314-24-s0001.docx]

**Supplemental Figures for:**

Diverse and unconventional methanogens, methanotrophs, and methylotrophs in metagenome-assembled genomes from subsurface sediments of the Slate River floodplain, Crested Butte, CO USA

Anna N. Rasmussen^a^, Bradley B. Tolar^a^*, John R. Bargar^b^, Kristin Boye^c^, and Christopher A. Francis^a,d^#

**Figure S1** Number of MAGs generated for each phylum colored by depth including both OBJ1 and OBJ2 sites together. Co-assemblies are indicated as the combined depths used (i.e., 70/90/110 include samples from 70, 90, and 110cm).

**Figure S2** RPKG (reads recruited per kilobase of genome per gigabase of metagenome) abundance of dereplicated (98% ANI) C1-cycling A) archaea MAGs, including methanogens, methanotrophs, and putative methylotrophs, and B) methylotrophic bacteria MAGs.

**Figure S3** Methanogenesis, methylotrophy, and other genes of interest encoding key proteins found in dereplicated methanogen, and archaeal methanotroph/methylotroph MAGs. ACD, acetate-CoA ligase; *rbcL*/*cbbL*, ribulose-bisphosphate carboxylase large chain; PRK/*prkB*, phosphoribulokinase; rGlyP, reductive glycine pathway; *glyA*/SHMT, glycine hydroxymethyltransferase; GCV, glycine cleavage system; RuMP, ribulose monophosphate cycle; *hxlB*, 6-phospho-3-hexuloisomerase; *hps-phi*, 3-hexulose-6-phosphate synthase / 6-phospho-3-hexuloisomerase; *ttuC*/*dmlA*, tartrate dehydrogenase/decarboxylase / D-malate dehydrogenase; ME2/*sfcA*/*maeA*/*mdh*, malate dehydrogenase; *leuB*/IMDH, 3-isopropylmalate dehydrogenase; *ech,* energy-converting hydrogenase; *hdrABC*, soluble heterodisulfide reductase; *hdrDE*, membrane bound heterodisulfide reductase; *mcr*, methylcoenzyme M reductase; *mtr*, tetrahydromethanopterin S-methyl-transferase; *mvh*, F420-non-reducing hydrogenase; *btu*, vitamin B12 transport system; *fds/fdo/fdh* formate dehydrogenase; *mttB*, trimethylamine methyltransferase; *mtmB,* methylamine methyltransferase; *mtgB*, glycine betaine methyltransferase; *mcp*, methyl-accepting chemotaxis protein; *che*, chemotaxis protein; *fla*, archaeal flagellar protein; CFAP65, cilia- and flagella-associated protein 65; *pil*, type IV pilus assembly protein; SOD, Fe-Mn family superoxide dismutase; *katG*, catalase-peroxidase; *katE*, catalase; *dfx*, superoxide reductase; CODH-ACS, carbon monoxide dehydrogenase/acetyl-CoA synthase; *acs*, acetyl-CoA decarbonylase/synthase; *cdh/cooFS*, anaerobic carbon-monoxide dehydrogenase; H4F, tetrahyrofolate; MTHFD, methylenetetrahydrofolate dehydrogenase; *metF*/MTHFR, methylenetetrahydrofolate reductase; *folD*, methylenetetrahydrofolate dehydrogenase; *fhs*, formate-tetrahydrofolate ligase; *fchA*, formate-tetrahydrofolate ligase; *tfx,* fmd operon transcriptional regulator; *mtd*, methylenetetrahydromethanopterin dehydrogenase; *mer*, 5,10methylenetetrahydromethanopterin reductase; *mch*, methenyltetrahydromethanopterin cyclohydrolase; *fwdA-F/fmdA-F*, formylmethanofuran dehydrogenase; *ftr*, formylmethanofuran-tetrahydromethanopterin N-formyltransferase.

**Figure S4** Select environmental data for locations sampled.

**Figure S5** ClustalOmega alignment of full-length copper monooxygenase protein sequences. Alignment was masked for gaps in > 80% of sequences. RAxML tree with 100 bootstraps. “Bin18*”* genomes highlighted in hot pink, *Ca*. Binataceae genomes in light pink. Archaeal *amoA* was used as the outgroup.

**Figure S6** Select genes for key C1-cycling and nitrogen-cycling proteins encoded in representative (dereplicated) MAGs from throughout the *Ca*. Binatia generated from Slate River floodplain sediments. ). ACD, acetate-CoA ligase; *ackA*, acetate kinase; ME2/*sfcA*/*mae*/*mdh*, malate dehydrogenase; *leuB*/IMDH, 3-isopropylmalate dehydrogenase; AGXT, alanine-glyoxylate transaminase; *pmo*, particulate methane monooxygenase; *cox*, carbon monoxide dehydrogenase; *fds/fdo/fdh/fdw* formate dehydrogenase; *xoxF*, lanthanide-dependent methanol dehydrogenase; *mauAB*, methylamine dehydrogenase; *nosZ*, nitrous oxide reductase; *nor*, nitric oxide reductase; *nirK*, nitrite reductase; *nar/nxr*, nitrate reductase/nitrite oxidoreductase; *nifHDK*, nitrogenase; *prm*, propane monooxygenase; *ladA*, long-chain alkane monooxygenase; dhaA, haloalkane dehalogenase; *alk*, alkane monooxygenase; CODH-ACS, carbon monoxide dehydrogenase/acetyl-CoA synthase; *acsA*, acetyl-CoA decarbonylase/synthase; H4F, tetrahyrofolate; MTHFD, methylenetetrahydrofolate dehydrogenase; *metF*/MTHFR, methylenetetrahydrofolate reductase; *folD*, methylenetetrahydrofolate dehydrogenase; *fhs*, formate-tetrahydrofolate ligase; *mer*, 5,10methylenetetrahydromethanopterin reductase; *mch*, methenyltetrahydromethanopterin cyclohydrolase; Fhc, formyltransferase/hydrolase complex; *fwdABC/fmdABC,* formylmethanofuran dehydrogenase

**Figure S7** Concatenated ribosomal tree including GTDB species representatives from the *Methyloceanibacter* genus and dereplicated MAGs from SR greater than 30% complete. Phylogeny made using a concatenated alignment of 20 ribosomal genes and IQ-TREE with model LG+F+R3 and 1000 bootstraps.

**
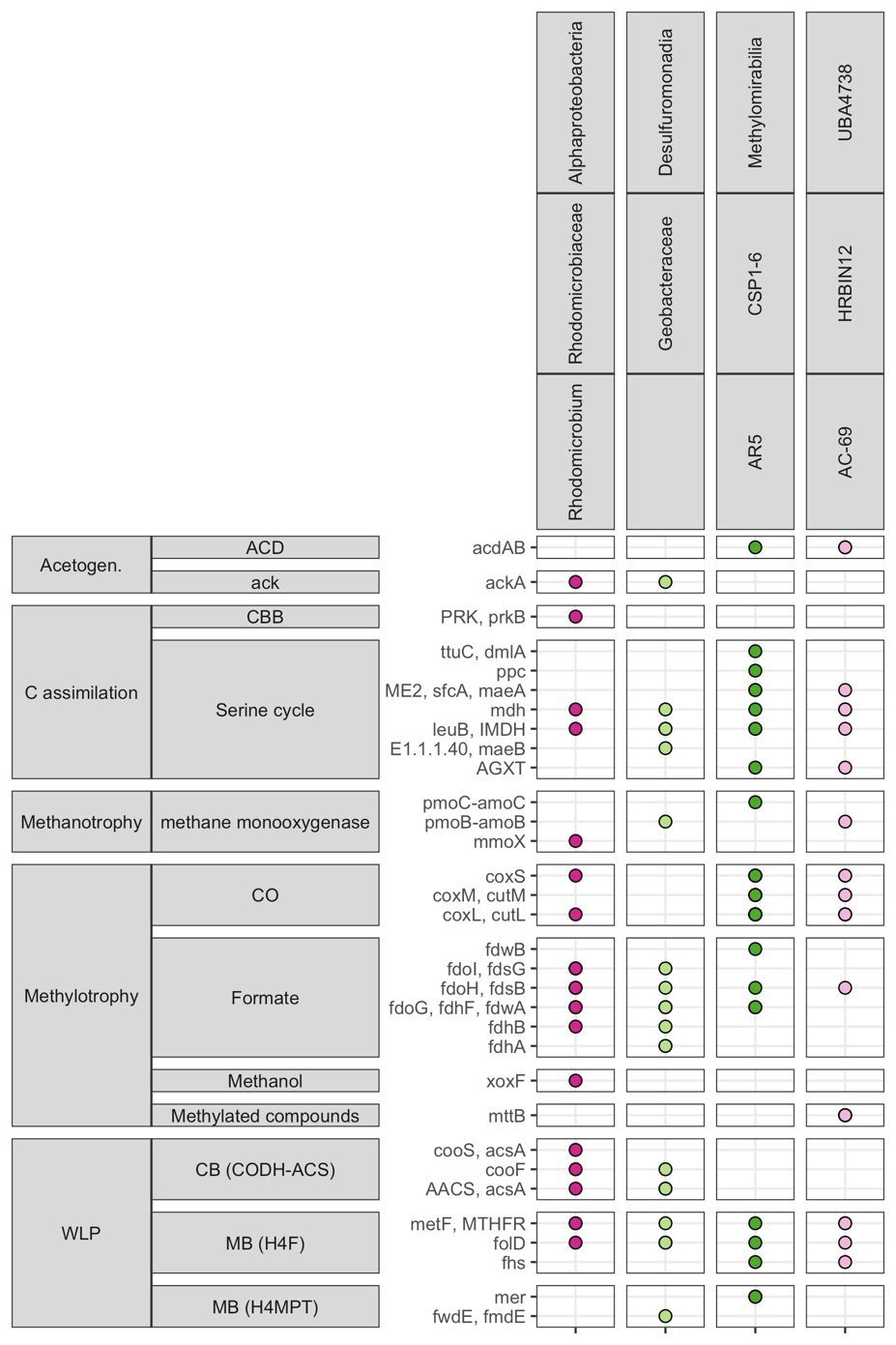
**

**Figure S8** Methylotrophy genes encoding for key proteins present in low-abundance MAGs with a single *pmo/mmo* gene. ACD, acetate-CoA ligase; *ackA*, acetate kinase; CBB, Calvin–Benson–Bassham cycle; PRK/*prkB*, phosphoribulokinase; ME2/*sfcA*/*mae*/*mdh*, malate dehydrogenase; *leuB*/IMDH, 3-isopropylmalate dehydrogenase; AGXT, alanine-glyoxylate transaminase; *mmo*, soluble methane monooxygenase; *pmo*, particulate methane monooxygenase*; cox*, carbon monoxide dehydrogenase; *fds/fdo/fdh/fdw* formate dehydrogenase; *xoxF*, lanthanide-dependent methanol dehydrogenase; *mttB*, trimethylamine methyltransferase; CODH-ACS, carbon monoxide dehydrogenase/acetyl-CoA synthase; *acs*, acetyl-CoA decarbonylase/synthase; *cdh/cooFS*, anaerobic carbon-monoxide dehydrogenase; H4F, tetrahyrofolate; *metF*/MTHFR, methylenetetrahydrofolate reductase; *folD*, methylenetetrahydrofolate dehydrogenase; *fhs*, formate-tetrahydrofolate ligase; *mer*, 5,10methylenetetrahydromethanopterin reductase; *fwdA-F/fmdA-F,* formylmethanofuran dehydrogenase

**
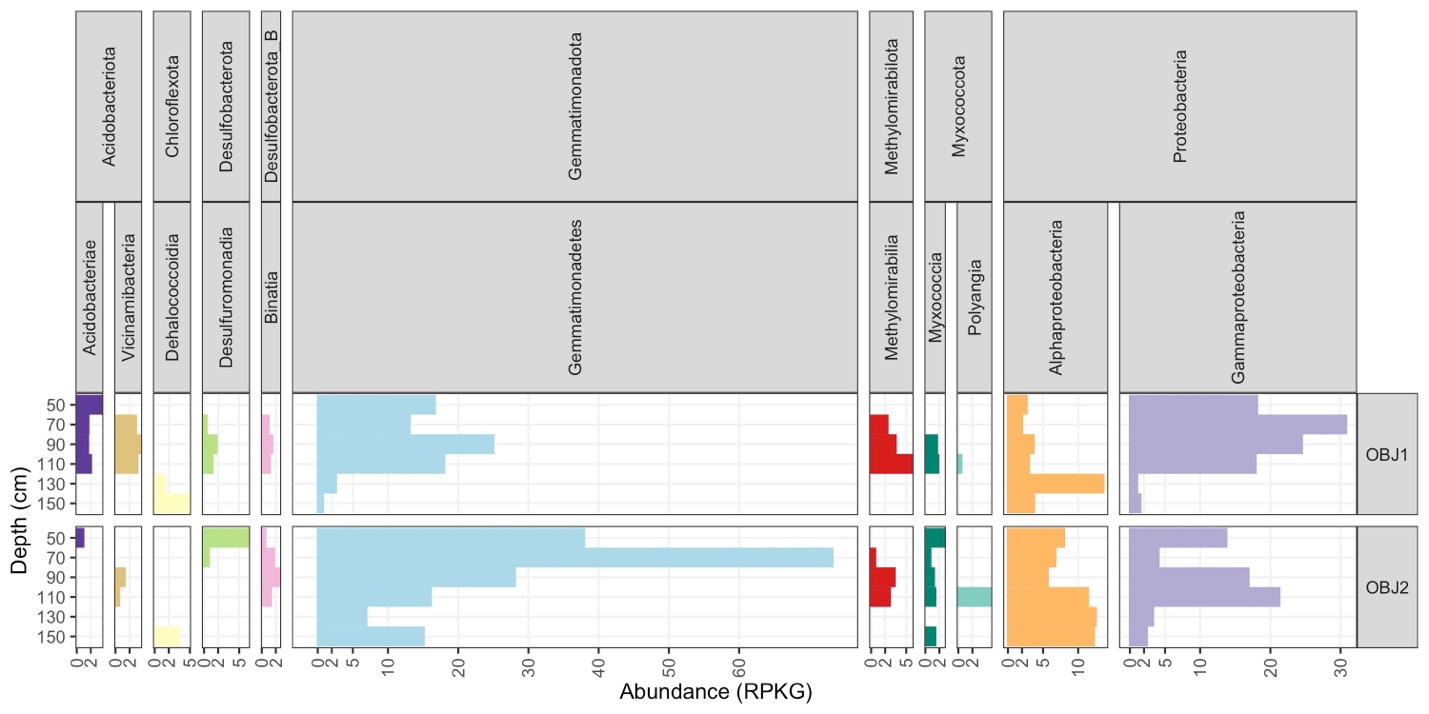
**

**Figure S9** Abundance of MAGs encoding methanol dehydrogenase.

**
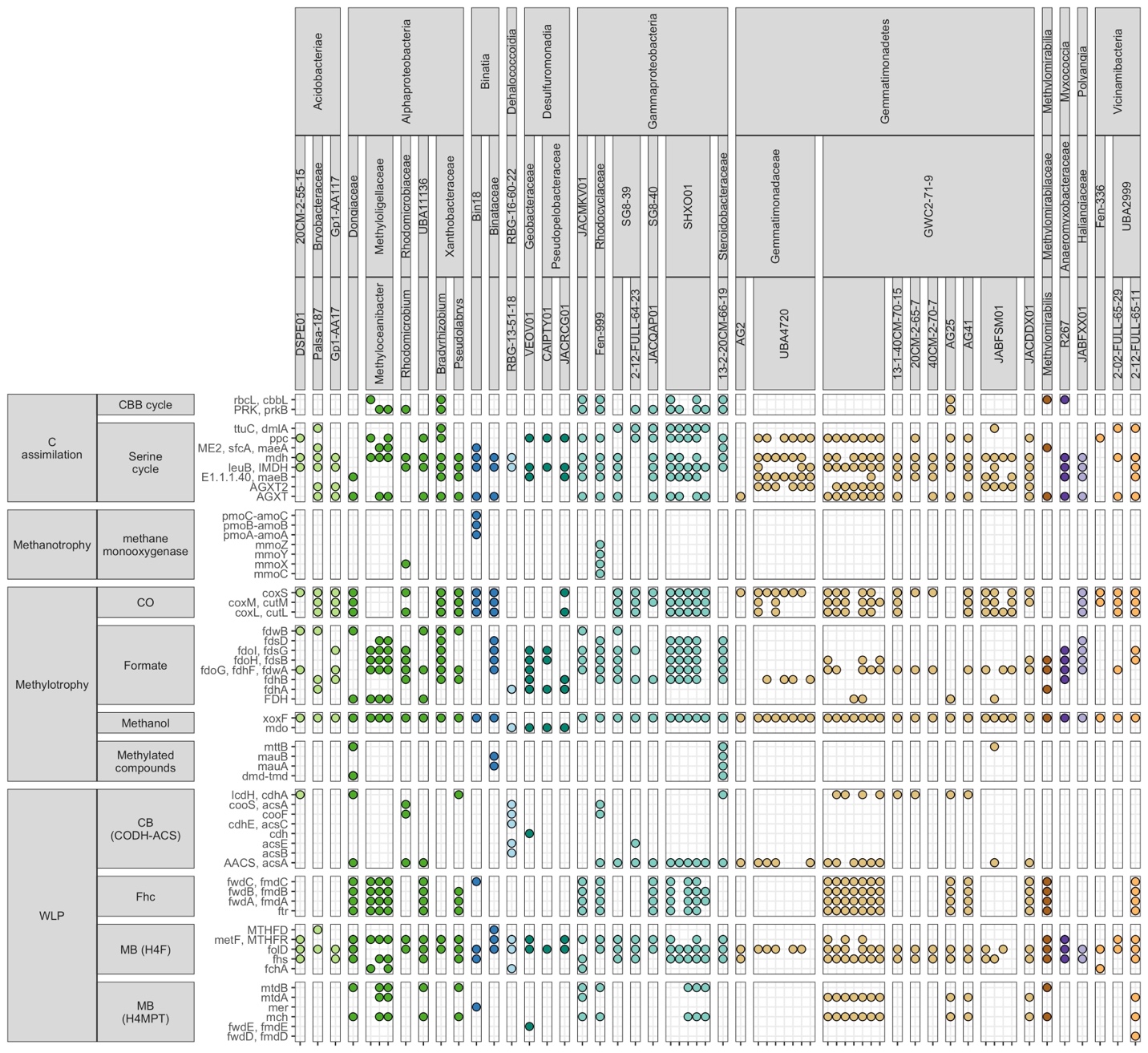
**

**Figure S10** Genes encoding key methylotrophy proteins present in MAGs containing a methanol dehydrogenase gene. CBB, Calvin–Benson–Bassham cycle; *rbcL*/*cbbL*, ribulose-bisphosphate carboxylase large chain; PRK/*prkB*, phosphoribulokinase; ME2/*sfcA*/*maeA*/*mdh*, malate dehydrogenase; *leuB*/IMDH, 3-isopropylmalate dehydrogenase; *mmo*, soluble methane monooxygenase; *pmo*, particulate methane monooxygenase; *cox*, carbon monoxide dehydrogenase; *fds/fdo/fdh* formate dehydrogenase; *xoxF*, lanthanide-dependent methanol dehydrogenase; *mdo*, methanol dehydrogenase; *mttB*, trimethylamine methyltransferase *mauAB*, methylamine dehydrogenase; *dmd-tmd*, dimethylamine/trimethylamine dehydrogenase; CODH-ACS, carbon monoxide dehydrogenase/acetyl-CoA synthase; *acs*, acetyl-CoA decarbonylase/synthase, *cdh*/*cooFS*; anaerobic carbon-monoxide dehydrogenase; H4F, tetrahyrofolate; MTHFD, methylenetetrahydrofolate dehydrogenase; *metF*/MTHFR, methylenetetrahydrofolate reductase; *folD*, methylenetetrahydrofolate dehydrogenase; *fhs*, formate-tetrahydrofolate ligase; *fchA*, formate-tetrahydrofolate ligase; *mtd*, methylenetetrahydromethanopterin dehydrogenase; *mer*, 5,10methylenetetrahydromethanopterin reductase; *mch*, methenyltetrahydromethanopterin cyclohydrolase; Fhc, formyltransferase/hydrolase complex; *fwdABC/fmdABC,* formylmethanofuran dehydrogenase; *ftr*, formylmethanofuran-tetrahydromethanopterin N-formyltransferase

**Figure S11** Genes encoding key methane/C1-cycling proteins in representative *Bathyarchaeia*, *Thermoplasmata, Methanomicrobia,* and *Methanosarcinia* MAGs. ACD, acetate-CoA ligase; *ackA*, acetate kinase; CBB, Calvin–Benson–Bassham cycle; *rbcL*/*cbbL*, ribulose-bisphosphate carboxylase large chain; PRK/*prkB*, phosphoribulokinase; rGlyP, reductive glycine pathway; *glyA/SHMT*, glycine hydroxymethyltransferase; *gcv*, glycine cleavage system; RuMP, ribulose monophosphate cycle; *hxlB*, 6-phospho-3-hexuloisomerase; *hps-phi*, 3-hexulose-6-phosphate synthase / 6-phospho-3-hexuloisomerase; *ttuC/dmlA*, tartrate dehydrogenase/decarboxylase / D-malate dehydrogenase; *ME2/sfcA/maeA/mdh*, malate dehydrogenase; *leuB/IMDH*, 3-isopropylmalate dehydrogenase; *ech,* energy-converting hydrogenase; *hdrABC*, soluble heterodisulfide reductase; *hdrDE*, membrane bound heterodisulfide reductase; *mcr*, methylcoenzyme M reductase; *mtr*, tetrahydromethanopterin S-methyl-transferase; *mvh*, F420-non-reducing hydrogenase; *cox*, carbon monoxide dehydrogenase; *btu*, vitamin B12 transport system; *fds/fdo/fdh* formate dehydrogenase; *mtaB*, methanol-5-hydroxybenzimidazolylcobamide co-methyltransferase; *mttC*, trimethylamine corrinoid protein; *mttB*, trimethylamine methyltransferase; *mtmB,* methylamine methyltransferase; *mtgB*, glycine betaine methyltransferase; CODH-ACS, carbon monoxide dehydrogenase/acetyl-CoA synthase; *acs*, acetyl-CoA decarbonylase/synthase, *cdh*/*cooFS*; anaerobic carbon-monoxide dehydrogenase; H4F, tetrahyrofolate; MTHFD, methylenetetrahydrofolate dehydrogenase; *metF*/MTHFR, methylenetetrahydrofolate reductase; *folD*, methylenetetrahydrofolate dehydrogenase; *fhs*, formate-tetrahydrofolate ligase; *fchA*, formate-tetrahydrofolate ligase; *tfx,* fmd operon transcriptional regulator; *mtd*, methylenetetrahydromethanopterin dehydrogenase; *mer*, 5,10methylenetetrahydromethanopterin reductase; *mch*, methenyltetrahydromethanopterin cyclohydrolase; *fwdA-F/fmdA-F*, formylmethanofuran dehydrogenase; *ftr*, formylmethanofuran-tetrahydromethanopterin N-formyltransferas
